# Object recognition ability predicts category learning with medical images

**DOI:** 10.1186/s41235-022-00456-9

**Published:** 2023-02-01

**Authors:** Conor J. R. Smithson, Quentin G. Eichbaum, Isabel Gauthier

**Affiliations:** 1 Department of Psychology, Vanderbilt University, PMB 407817, 2301 Vanderbilt Place, Nashville, TN 37240-7817 USA; 2 Department of Pathology, Microbiology and Immunology, Vanderbilt University, Nashville, USA; 3 Vanderbilt Pathology Education Research Group, Nashville, USA

**Keywords:** Category learning, Categorisation, Object recognition, Individual differences, Medical images, Radiology

## Abstract

We investigated the relationship between category learning and domain-general object recognition ability (*o*). We assessed this relationship in a radiological context, using a category learning test in which participants judged whether white blood cells were cancerous. In study 1, Bayesian evidence negated a relationship between *o* and category learning. This lack of correlation occurred despite high reliability in all measurements. However, participants only received feedback on the first 10 of 60 trials. In study 2, we assigned participants to one of two conditions: feedback on only the first 10 trials, or on all 60 trials of the category learning test. We found strong Bayesian evidence for a correlation between *o* and categorisation accuracy in the full-feedback condition, but not when feedback was limited to early trials. Moderate Bayesian evidence supported a difference between these correlations. Without feedback, participants may stick to simple rules they formulate at the start of category learning, when trials are easier. Feedback may encourage participants to abandon less effective rules and switch to exemplar learning. This work provides the first evidence relating *o* to a specific learning mechanism, suggesting this ability is more dependent upon exemplar learning mechanisms than rule abstraction. Object-recognition ability could complement other sources of individual differences when predicting accuracy of medical image interpretation.

## Introduction

Accurate interpretation of medical images plays a crucial role in the diagnosis of many medical conditions. This process often requires the visual detection of abnormalities, such as lung nodules in radiographs or masses in mammograms. Although experts undergo substantial training, they cannot always make the correct decision (Brady, 2017; Graber et al., 2002). For many diagnostic tests, there are substantial discrepancies in accuracy between practitioners, in part due to differences in experience (Itani et al., 2019; Rudolph et al., 2021). Practitioners may even disagree with their own initial judgement when asked to review images a second time (Abujudeh et al., 2010). Although precise estimates of the prevalence of medical imaging errors are difficult to obtain, as errors vary widely based on test, practice setting, and population, estimates of real-world error rates range from < 1% to around 10% (Gergenti & Olympia, 2019; Lamoureux et al., 2021; Lockwood, 2017). Error rates can be higher still when the relevant disease is rare in the studied population (Kolb et al., 2002). These errors have multiple causes at the individual and the system level, including fatigue, communication failure, biased reasoning, failures of visual search, interpretive errors, technological errors, and poor technique (Lee et al., 2013; Waite et al., 2017). A majority of radiological errors are perceptual in nature, with practitioners failing to spot abnormalities, whereas a smaller but still substantial proportion of errors are due to failure to correctly categorise abnormalities (Donald & Barnard, 2012; Ferguson et al., 2021; Kim & Mansfield, 2014).

As the accurate interpretation of medical images relies on the detection and categorisation of objects, differences in diagnostic accuracy among practitioners may partially result from individual differences in visual abilities. The existence of such differences is supported by evidence for a domain-general object recognition ability (*o*). Confirmatory factor models demonstrate that diverse measures of object recognition, with differing task demands and differing object categories, load onto a single higher-order factor (Richler et al., 2019). The *o* factor explains variance in scores on object recognition tests beyond that explained by intelligence and visual working memory, and it can do so for both familiar and unfamiliar object categories (Richler et al., 2017; Sunday et al., 2022). In studies that are not concerned with investigating the structure of this visual ability, or that for practical reasons cannot achieve the sample size or the number of tasks required for structural equation modelling, an aggregate approach (Rushton et al., 1983) to measuring object recognition ability has been used (Chang & Gauthier, 2021; Chow et al., 2022). In this approach, *z*-scores on two object recognition tests that differ in format and stimuli are averaged to estimate the level of the underlying *o* ability. This approach provides a valid compromise in estimating *o* in smaller samples and when time is limited (Smithson et al., 2022). Using this approach, Sunday et al. (2018) found that *o* predicts the accurate detection of lung nodules in chest radiographs for both novices and experts, demonstrating a link between *o* and successful abnormality detection. The detection of lung nodules depends on successful visual search, but other radiological tasks rely less on visual search, and more on accurate categorisation.

As *o* captures the ability to learn individual identities, it is unclear whether it will also predict accurate categorisation. There are demonstrated individual differences in both speed and accuracy of category learning, in addition to differences in strategy use. Some people rely more on the abstraction of simple rules, leading to categorisation decisions based on one dimension. Others preferentially rely on judgements of perceptual similarity to category exemplars, which can be measured in a space defined by several relevant dimensions (Little & McDaniel, 2015; Wahlheim et al., 2016). For example, one may learn to categorise a skin mole as cancerous if it is asymmetrical, but one may also rely on comparisons of the mole to remembered examples of cancerous and non-cancerous moles. *o* predicts performance on many visual tasks that require judgements other than individuation, such as visual search, and judgements of summary statistics for ensembles (Chang & Gauthier, 2021; Sunday et al., 2018). Given that *o* can predict such a wide array of visual tasks, it is reasonable to question whether *o* could also predict accurate categorisation in a visual domain. There is some support for a relationship between individual differences in object recognition (measured by one of the tasks that tap into *o*) and accurate categorisation of medical images, at least under some conditions. In one study, participants categorised white blood cells as cancerous or not under conditions emphasising speed or accuracy, or when provided with a biased cue (Trueblood et al., 2018). Performance on an object recognition test predicted accurate categorisation, particularly for categorisation following biased cues. While this suggests that categorisation may rely on visual abilities under some conditions more than others, this work did not have sufficient power to compare correlations across conditions.

The study of domain-general high-level visual abilities is an emerging research area (Gauthier, 2018; Gauthier et al., 2022), and the extent to which these abilities can explain variability in performance on real-world tasks is still unclear. As diagnostic imaging has a heavy visual component, it is plausible that visual abilities may influence performance on these tasks. A good first step in showing this is to demonstrate that *o* can predict accurate categorisation of medical images. To investigate this, we created a three-alternative forced choice test in which participants learn to categorise white blood cell images as cancerous (blast) or non-cancerous (non-blast). We used a novice sample to test the relationship between *o* and categorisation in the absence of extensive pre-existing experience, which could contaminate the relationship.

## Study 1

### Participants

Thirty-nine Vanderbilt University students participated for course credit. A further sixty-seven adults were recruited on Amazon Mechanical Turk. Recruitment criteria required the use of a US IP address, greater than 50 approved hits, and a greater than 90% approval rating. We used a Bayesian stopping rule, collecting data in batches, until the Bayes Factor for the correlation between *o* and performance on the Blast Test reached a suggested threshold for moderate evidence, BF_10_ > 3 or BF_10_ < 1/3 (Lee & Wagenmakers, 2014). From our total of 106 participants, we excluded 26 for below-chance performance on either of the two tests used for estimating *o*.^1^ This left 80 participants in the final analysis (*M*age = 34.7, *SD* = 14.8; 32 men, 46 women, 2 other).

### Materials and procedure

**Fig. 1:**
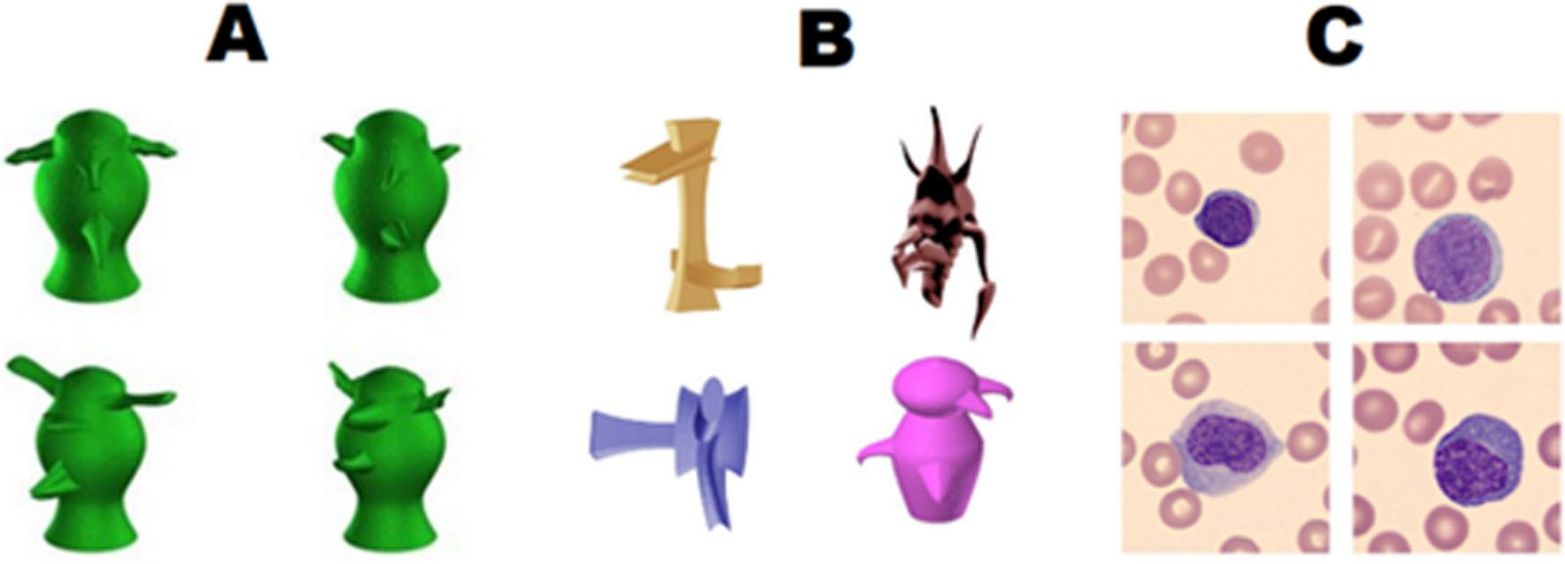
Example Stimuli. **A:** Symmetrical Greebles used in the NOMT. **B:** Novel objects used in the Object Matching Test. From the top left, anticlockwise: vertical and horizontal Ziggerins, asymmetrical Greebles, and Sheinbugs. **C:** White blood cells used in the Blast Test

Participants completed three on-screen tests. First, they completed the Blast Test—a category learning test involving the identification of cancerous cells. After this, they completed the Novel Object Memory Test (NOMT) and the Object Matching Test, which were used to estimate *o*. Example stimuli for all three tests can be seen in Fig. 1. We used a fixed order of trials for all tests to minimise variance due to factors other than individual differences. Informed consent was obtained from all participants, and the study was approved by the Vanderbilt University Institutional Review Board.

### Development of Blast Test

We obtained images of blast and non-blast blood cells from peripheral blood smears conducted at Vanderbilt University Medical Center. These images have been used in prior research on medical decision making (Hasan et al., 2021; Trueblood et al., 2018). They were categorised by expert consensus as blast or non-blast. They were additionally sorted into easy or hard categories on the basis of whether each cell image shared features common to the other category (Trueblood et al., 2018). We initially created 100 trials. Each trial was composed of two non-blast cells, and one blast cell. The task on each trial was to identify the blast cell from a side-by-side display of the three images. The use of three cells to choose from on each test trial reduces the importance of response bias and reduces the successful random guessing rate for each trial, compared to using only two. We initially created 25 trials composed of one easy blast image, and two easy non-blast images; 25 trials composed of one easy blast and two hard non-blast images; 25 trials composed of one hard blast and two easy non-blast images; and 25 trials composed of one hard blast, and two hard non-blast images. On the basis of pilot testing, we selected trials from a broad range of difficulty levels to maximise the informativeness of our test across a wide range of ability levels. Trials were also selected for high reliability; we checked item-rest correlations, and internal consistency if an item was dropped, and dropped those that reduced reliability. In the final Blast Test, the trials were ordered from easiest to hardest based on our pilot data. To familiarise participants with the two categories, participants were first shown 6 blast blood cell images, and 6 non-blast blood cell images, with category membership clearly labelled. Participants then completed 60 trials. Feedback indicating whether responses were correct appeared at the top of the screen for 1 s after each of the first 10 trials. No feedback was given for the remaining 50 trials. Percent correct over the 60 trials indexed performance.

### Tests to estimate *o*

As in prior work, we used the aggregate of two object recognition tests to estimate *o* (e.g. Chang & Gauthier, 2021; Chow et al., 2022; Sunday et al., 2018). These two tests were chosen from a battery of tests that were good indicators of *o* in confirmatory factor models (Richler et al., 2019; Sunday et al., 2022), on the basis that they have different test constraints and use different categories of novel objects. The aggregation of scores from tests using different object categories and different task demands purifies the measurement of domain-general ability, by reducing the proportion of variance in scores that is due to irrelevant variation specific to particular task demands or stimuli (Rushton et al., 1983). The expected correlation for a pair of such tests is relatively low (0.3–0.4) because superficial features of the tests and stimuli are different. The aggregate of standardised performance on two tests provides a good estimate (*r* ≈ 0.8) of *o* measured as a factor score in a confirmatory factor analysis based on six tests (Smithson et al., 2022).

### Novel object memory test

The NOMT was developed to assess object recognition ability (Richler et al., 2017). Participants were asked to memorise six exemplars from a category of novel objects (symmetrical Greebles; Gauthier & Tarr, 1997). They then viewed these six targets for as long as they needed, before completing six test trials. On each test trial, one target Greeble appeared alongside two distractor Greebles. Participants had unlimited time to select the target Greeble with their mouse on each trial. Participants then reviewed the targets and completed a further 18 test trials. Participants were then informed that the Greebles could appear in different viewpoints on remaining trials. The targets were presented again for review, prior to the final 24 test trials. Percent correct over the 48 test trials indexed performance.

### Object matching test

On each trial participants had to determine whether two serially presented images displayed the same object. The objects were selected from four categories of novel objects: asymmetrical Greebles, Sheinbugs, and two distinct categories of Ziggerins (Richler et al., 2019). Each trial used either one or two objects from the same category. Each trial began with the presentation of a central fixation cross for 500 ms. The target object was then presented for 300 ms before a visual mask composed of scrambled object parts appeared for 500 ms. Finally, another object was presented which was either the same as the target or different. Participants had four seconds to respond by clicking either the same or different buttons on-screen. The target object could change in orientation or size from study to test, but participants were asked to judge only whether the identity of the object was the same. After an initial four practice trials, participants completed 70 test trials. Performance was indexed by a signal detection theory measure of sensitivity (*d*′). Timed-out responses were not included in the calculation. Less than 1% of all trials had timed-out responses.

### Results

**Fig. 2:**
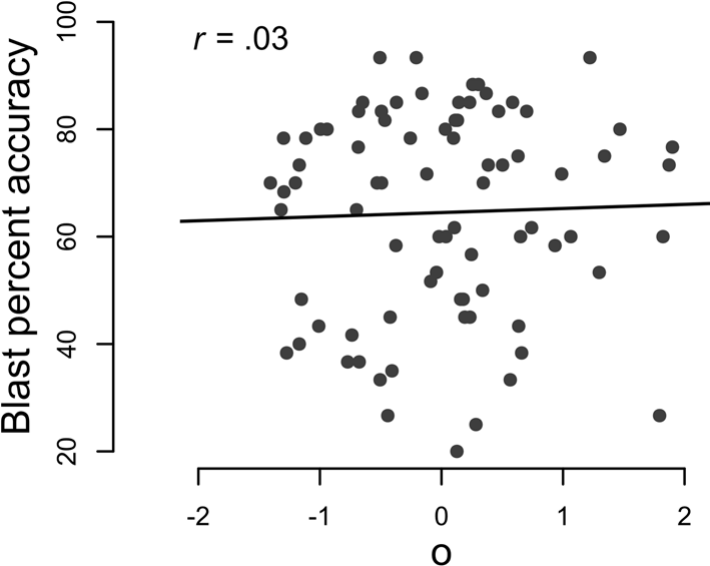
Correlation between *o* and percent accuracy on the Blast Test

Descriptive statistics and reliability for each test can be seen in Table 1. To estimate *o, z*-scores for percent accuracy on the NOMT and *d*′ on the Object Matching Test were averaged. Correlational analyses used a Jeffreys-beta prior (Jeffreys, 1961) with a scale of 1 and were conducted with the BayesFactor Package (Morey & Rouder, 2021) in R. BF_+0_ indicates a one-sided test in the positive direction, and BF_10_ is used for two-sided tests. We report highest posterior densities as 95% credibility intervals, and the median of the posterior distribution is used for parameter estimation. Our reported CIs and parameter estimates are always calculated from two-sided analyses. As expected, there was very strong Bayesian evidence for a positive correlation between performance on the NOMT and the Object Matching Test (*r* = 0.33, 95% CI [0.13, 0.52], BF_+0_ = 31.07). We obtained moderate evidence against a correlation between *o* and percent accuracy on the Blast Test (*r* = 0.03, 95% CI [− 0.18, 0.25] BF_+0_ = 0.18, Fig. 2), although this was somewhat sensitive to the choice of prior, with the Bayes factor rising above 1/3rd for prior scales equalling or below 0.32.

**Table 1:** Descriptive statistics

Test	Mean (*SD*)	Reliability
NOMT (percent accuracy)	55.4% (15.2%)	0.75
Object Matching (*d*′)	0.98 (0.53)	0.73
*o*	0 (0.82)	0.81
Blast (percent accuracy)	64.5% (19.1%)	0.93

Reliability is calculated using Pearson’s *r* between two halves composed of alternating trials, with the Spearman–Brown prophecy formula applied. Aggregate reliability of *o* was calculated with equal weighting using a formula adapted from Wang and Stanley (1970)

### Discussion

Contrary to our hypothesis, *o* did not predict performance on the Blast Test. One possible reason for the lack of a relationship with category learning may be the limited amount of feedback that participants received. Although tests that are used to estimate *o* do not use feedback, and *o* can predict other skills measured in tests without feedback, such as working memory judgements with musical notation (Chang & Gauthier, 2021) or food oddball judgements (Gauthier & Fiestan, 2023), the strategies and mechanisms recruited during category learning may be particularly sensitive to feedback. Early on in category learning, people tend to rely on simple rule-based judgements and then update these rules as they receive further feedback, before shifting to similarity-based exemplar retrieval as expertise develops (Johansen & Palmeri, 2002). In Study 1, we only provided participants with feedback on the first ten trials of the Blast Test. As earlier trials in the Blast Test are easier, participants did not receive any feedback on more difficult trials. Individual differences in performance may thus result from divergent initial rule choices, or differing success in the application of these rules. Due to the limited feedback, participants may have seen no need to update their initial rules or may have had no basis on which to do so. Additionally, the lack of feedback may have discouraged a switch in strategy to reliance on judgements of perceptual similarity to prior exemplars. Harder trials are presumably more likely to require methods of judgement other than simple rule use. The tests used to estimate *o* require within-category individuation, which also cannot usually rely on the use of simple rules, as objects in a common category will share a basic configuration of parts.

To test whether the lack of association between *o* and Blast Test accuracy was due to the limited feedback, we repeated the study with the addition of a full-feedback condition, wherein participants received feedback for all 60 trials of the Blast Test.

## Study 2

### Materials and procedure

Participants completed the same three tests as in study one. However, the tests were in a different fixed order: NOMT, Object Matching Test, and Blast Test. In Study 2, we compared the limited feedback and the full-feedback versions of the Blast Test, so placing this test last ensured that performance on the two object recognition tests could not be affected by assignment to either condition of the Blast Test. The NOMT was modified such that participants had a fixed 20 s to familiarise themselves with the six targets on each study trial, reducing differences in study time as a source of individual differences. The Object Matching Test was altered so that for the first 35 trials the study object was presented for 600 ms. This was done to lower difficulty on some trials, as mean *d*′ was low in Study 1 (0.97). For the remaining 35 trials, the study object was presented for 300 ms, as in Study 1. Another alteration was to allow unlimited time to respond, eliminating timed-out responses so that *d*′ was calculated for the exact same trials for all participants.

### Participants

Due to the high percentage of participants excluded in Study 1 for below-chance performance (27.4%), we switched recruitment platform to Prolific.co. We recruited 245 participants, with the requirement of English fluency. Our pre-set exclusion criteria excluded one participant who failed more than one of five attention checks spread throughout the study. This method of exclusion allowed us to treat low scores as valid. The attention checks were dummy trials in which participants were instructed to click on a specific response option. Participants were randomly assigned to the limited or the full-feedback condition. Due to error, the first 6 participants were non-randomly assigned to the limited feedback condition. Once we reached 122 participants in the limited feedback condition (64 men, 57 women, 1 other; *M*age = 25.4, *SD* = 6.3), we added 21 participants to the full-feedback condition (50 men, 67 women, 5 other; *M*age = 26.3, *SD* = 8.8) to achieve equal group sizes (which were unequal due to random assignment as well as the initial error). We then ceased collecting data as we were able to find a conclusive Bayes factor for the existence of a correlation between *o* and categorisation accuracy in the full-feedback condition.

### Results

**Fig. 3:**
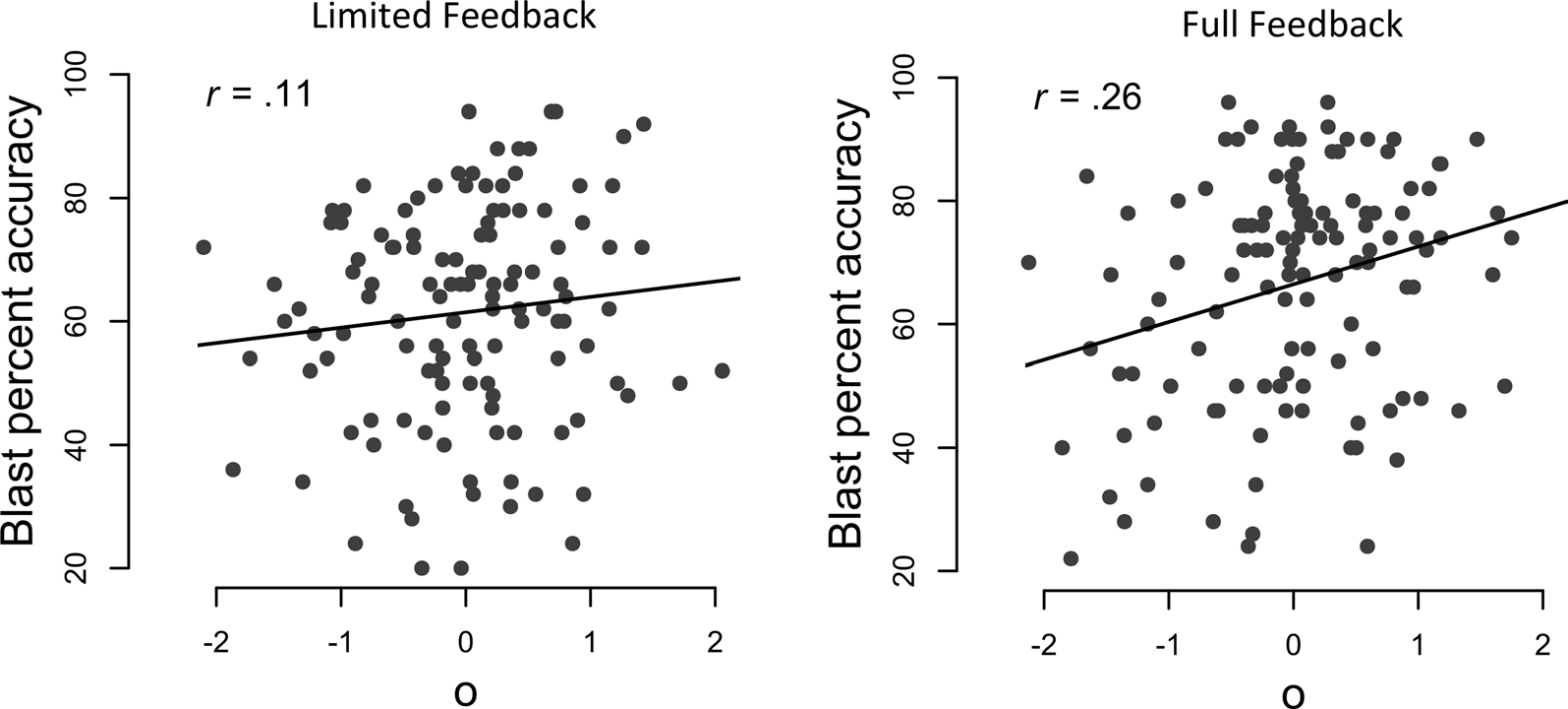
Correlation between o and percent accuracy on the Blast Test

Descriptive statistics and reliability for each test can be seen in Table 2. As expected, there was a correlation between the NOMT and the Object Matching Test (*r* = 0.26, 95% CI [0.14, 0.37], BF_+0_ = 752.82) across all participants. There was inconclusive evidence for an overall difference in accuracy on the last 50 trials of the Blast Test (conditions diverge after the first 10 trials) between the limited and the full-feedback conditions (BF_+0_ = 2.48). For the limited feedback condition, there was inconclusive evidence against a correlation between *o* and percent accuracy on the Blast Test (*r* = 0.11, 95% CI [− 0.07, 0.28], BF_+0_ = 0.41, Fig. 3.). For the full-feedback condition, there was strong evidence for a correlation between *o* and percent accuracy on the Blast Test (*r* = 0.26, 95% CI [0.10, 0.43], BF_+0_ = 18.75, Fig. 3.). Using BFpack (Mulder et al., 2019) in R, we obtained moderate Bayesian evidence for the hypothesis that the correlation between *o* and blast percent accuracy is greater in the full-feedback condition than in the limited feedback condition, compared to its complement (BF_10_ = 8.34).

**Table 2:** Descriptive statistics

	Limited feedback		Full feedback	
Test	Mean (*SD*)	Reliability	Mean (*SD*)	Reliability
NOMT (percent accuracy)	59.72% (15.76%)	0.82	57.07% (14.24%)	0.76
Object Matching (*d*′)	1.44 (0.6)	0.81	1.33 (0.6)	0.83
*o*	0 (0.77)	0.85	0 (0.81)	0.84
Blast (percent accuracy)	61.46% (17.62%)	0.89	66.46% (18.49%)	0.92

Reliability is calculated using Pearson’s *r* for two halves composed of alternating trials, with the Spearman–Brown prophecy formula applied. Blast Test analyses here are of the last 50 trials only. Aggregate reliability of *o* was calculated with equal weighting using a formula adapted from Wang and Stanley (1970)

### Discussion

In support of our main hypothesis, we found a relationship between *o* and accuracy in the full-feedback condition of the Blast Test. We further showed that this relationship was greater than the relationship between *o* and Blast Test accuracy in the limited feedback condition. This suggests a relationship between *o* and category learning. We hypothesised that such a correlation would emerge in the full-feedback condition because greater feedback may allow for a shift from the use of simple rules for categorisation judgements to the use of complex rules, or a change in strategy from rule-based judgements to judgements based on perceptual comparison of cells against prior exemplars. However, because each trial included one blast image, and two non-blast images, one task-specific strategy that participants could have employed is to select the odd one out. Perhaps particularly for the limited feedback condition, participants may have used this strategy instead of trying to learn to categorise blast cells explicitly. To test this possibility, in study 3 we assessed participants in a no-feedback version of the Blast Test.

## Study 3

### Participants and method

Fifty-one participants (*M*age = 24.69, *SD* = 6.8; 24 men, 24 women, 3 other) were recruited on the Prolific.co platform, with a requirement for English fluency. We then assessed if our stopping criteria had been met, which was a Bayes factor < 1/3 or > 3 for a correlation between *o* and Blast Test accuracy. No participants were excluded, as none missed more than one of five attention checks. Participants completed the same tests in the same order as in Study 2. The only differences were that the Blast Test gave no feedback on performance, and no examples of blast and non-blast cells were given prior to testing. Participants were instructed to try their best to choose the cancerous cell on each trial, despite the lack of examples or feedback.

## Results and discussion

Descriptive statistics are presented in Table 3. Performance in the no-feedback condition of the Blast Test was slightly below-chance level (chance = 33%; *M* = 0.3, *SD* = 0.16), and we obtained strong Bayesian evidence from a one-sample *t-*test that performance is not above chance (BF_+0_ = 0.06). It appears that the presentation of a small number of examples and the presence of limited feedback are required for most people to perform above chance. Given the high performance in Study 1 and 2, it is unlikely that participants in those studies made use of an odd-one-out strategy. There was no correlation between *o* and accuracy in this version of the Blast Test (*r* = − 0.01, 95% CI [− 0.28, 0.25], BF_+0_ = 0.16).

**Table 3:** Descriptive statistics

Test	Mean (*SD*)	Reliability
NOMT (percent accuracy)	59% (15%)	0.79
Object Matching (*d*′)	1.24 (0.71)	0.85
*o*	0 (0.72)	0.83
Blast (percent accuracy)	30% (16%)	0.90

Reliability is calculated using Pearson’s *r* for two halves composed of alternating trials, with the Spearman–Brown prophecy formula applied. Blast Test here is the no-feedback version. Aggregate reliability of o was calculated with equal weighting using a formula adapted from Wang and Stanley (1970)

**Fig. 4:**
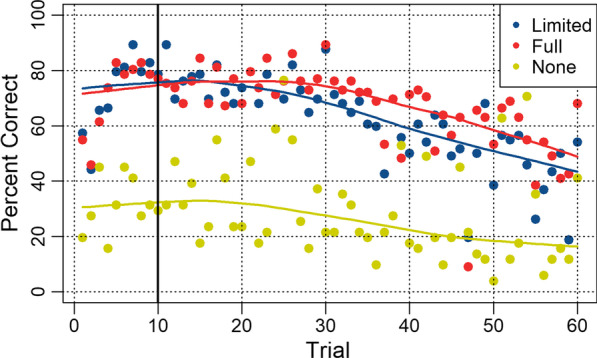
Percentage of participants who responded correctly per trial in Study 2 and 3. Note. Limited-feedback and full-feedback conditions are from Study 2, No-feedback condition is from Study 3. The solid vertical line indicates trial 10, after which feedback only continues for the full-feedback condition

To determine whether trial difficulty was consistent between the feedback conditions in Study 2 and the no-feedback condition in Study 3, we tested for cross-condition correlations for the percentage of participants who responded correctly on each trial. Figure 4 shows average accuracy per trial. To counter skewness, we applied a log transformation to the limited (− 0.75 to 0.32) and full (− 1.44 to − 0.15) feedback conditions, and a square root transformation to the no-feedback condition (0.82 to 0.25). There was a high correlation between trial accuracy in the full-feedback condition and the limited feedback condition (*r* = 0.83, 95% CI [0.74, 0.91], BF_10_ = 9.18 × 10^13^). There were much smaller correlations between trial accuracy in the no-feedback condition and the full feedback (*r* = 0.24, 95% CI [− 0.01, 0.48], BF_10_ = 0.94) and limited feedback (*r* = 0.31, 95% CI [0.08, 0.53], BF_10_ = 3.42) conditions.

Although the Blast Test was not designed to reveal strategy use, we suspected that participants who rely primarily on a simple rule may choose to use size as the basis and select the largest cell as being cancerous. We measured the maximum diameter of the cells in each trial, excluding small appendages (except in the case of ties), and found that size was diagnostic in nine of the first ten trials, and 70% of first-half trials, but only diagnostic in 33% of second-half trials.^2^ If participants in the limited feedback condition rely on a simple rule, it is likely that they would form a size-based rule, given that it is so diagnostic for early trials. Indeed, we found a strong correlation between percent accuracy on each trial and whether size was diagnostic in the no feedback (*r* = 0.56, 95% CI [0.37, 0.72], BF_+0_ = 26,208.3) and limited feedback conditions (*r* = 0.4, 95% CI [0.18, 0.60], BF_+0_ = 54.78), but a weaker one in the full-feedback condition (*r* = 0.31, 95% CI [0.08, 0.53], BF_+0_ = 7.43).

## General discussion

We found evidence in Study 2 that *o* predicts performance on a category learning task requiring the assessment of radiological images. This demonstrates a link between individual differences in individuation and categorisation, adding to a growing corpus of literature suggesting that individual differences in a wide variety of visual tasks are related (Chang & Gauthier, 2021, 2022; Growns et al., 2022). We also demonstrate in Study 1 and 2 that *o* either does not predict or at most predicts to a lesser extent, categorisation accuracy when participants receive feedback on only a very limited number of easy categorisation trials. This is despite the fact that performance in this limited feedback condition was very similar to performance in a condition where feedback was given continuously. Furthermore, participants who received limited feedback in Study 1 and 2 successfully categorised cells as cancerous or not with a success rate approximately double that of participants in Study 3, who received no feedback. In other words, we find the biggest difference in performance on the Blast Test between the no-feedback condition (Study 3) and any of the other conditions in which examples and some feedback were presented. This is not surprising given the extensive literature on the advantages of supervised learning for categories that are not based on a simple verbalisable rule (Ashby et al., 1999, 2002). In contrast, the difference between providing feedback only on ten trials vs. all trials was more modest in terms of average performance on the Blast Test. Nonetheless, this additional feedback made a substantial difference across individuals, leading to an advantage for those participants with higher *o*.

The correlation between *o* and performance in the full-feedback condition was small (*r* = 0.26, or *r* = 0.30 when accounting for attenuation from measurement error). The effect is similar to that in Sunday et al. (2018), who found a correlation of *r* = 0.28 between *o* and decisions on a test of tumour detection in chest radiographs, after controlling for intelligence. The correlation could be limited by the fact that the Blast Test is short and allows different strategies. But more importantly, *o* is conceived as a general ability that does not reflect specifics of the domain or the task constraints. Performance on any test is explained by a variety of factors, some of them general and some specific to the test. For instance, two similarly formatted matching tests may correlate more strongly (e.g. Growns et al., 2022), but some of this correlation may be due to specific task requirements. When tests with different formats use similar stimuli (e.g. faces, Wilmer et al., 2014), the resulting strong correlation is partially due to the common domain. But when two tests differ in both format and domain, like the tests we use to estimate *o,* the shared variance is expected to be smaller. Importantly, the advantage is that we can expect a domain-general ability to predict some of the variance in other very different tasks, such as the Blast categorisation test. This is somewhat analogous to intelligence, which is, for instance, a predictor of job performance in many domains, with effect sizes that are comparable to what we observe here (e.g. *r* = 0.33; see Ree & Earles, 1992, for a review). In addition, it is important to note that a small effect size when measured in a single task can translate into large consequences in the long run, in real-world situations where individuals make a very large number of perceptual decisions in the course of their work (Funder & Ozer, 2019).

By crossing the measurement of *o* with an experimental manipulation, our results are the first to speak to the mechanisms that support this ability, because of the extensive literature distinguishing different modes for category learning. One influential model proposes two systems for category learning, one using simple explicit rules, and the other for learning more complex multidimensional categories that are difficult to verbalise (Ashby et al., 1999). A different account suggests that even within a single system, feedback is more critical for more cognitively demanding categorisation tasks (Le Pelley et al., 2019). In the Blast Test, early category learning can plausibly rely primarily on simple rule generation. As difficulty increases, a simple rule will become ineffective and participants need to switch to judgements of similarity to stored exemplars. In summary, many different visual tasks tap into *o*, but this ability may not support categorisation following simple verbalisable rules, or categorisation of a multidimensional nature without mechanisms responsible for supervised learning. Fully supervised learning may not be necessary; semi-supervised learning (e.g. labelling a few exemplars) is the predominant method by which humans learn categories (Gibson et al., 2013; LaTourrette & Waxman, 2019), and we would also expect *o* to predict categorisation abilities that have developed through this method. These conjectures could be more directly addressed by testing for correlations between *o* and accuracy on categorisation tasks that only require simple rules, require a combination of rules to increase cognitive demands, or which require multidimensional judgements which cannot easily be reduced to verbalisable rules. To make strong claims about the underlying categorisation strategies being employed by individual participants would require an analysis of response patterns in tasks that have been designed to reveal strategy use.

The finding that *o* can predict successful categorisation adds to existing knowledge that *o* can predict successful visual search in a radiological task (Sunday et al., 2018). Both perceptual and interpretative skills are fundamental for radiological diagnostics. Therefore, *o* may plausibly predict diagnostic accuracy; although we have not yet tested this in an ecologically valid design. Accuracy on category learning experiments can be influenced by task demands and sequence effects, in which case, performance may reflect the successful use of task-specific strategies that may be hard to account for (Richler & Palmeri, 2014; Stewart et al., 2002). Nevertheless, when combined with the influences of experience and general intelligence, the contribution of *o* may provide a fuller explanation of individual differences in diagnostic accuracy. Further research should explore a relationship between *o* and categorisation accuracy in a radiological task using an expert sample. The contribution of *o* to performance and the conditions necessary for it to be used may well differ in experts. For instance, the contribution of *o* to categorisation may be greater for experts than for novices, because experts have more exemplars in memory and may rely less on simple categorisation rules. In addition, feedback may not be necessary for a relationship with *o* to emerge in experts who can already categorise blast from non-blast cells.

## Data Availability

Data, analysis code, and materials are available at https://osf.io/yaqs8/. No studies were preregistered.

## Footnotes

1 19 excluded participants were from the Mechanical Turk sample, and 7 were from the student sample.

2 Trial images are available at https://osf.io/yaqs8/.
